# Lectures on Some of the Recent Investigations into the Pathology of Infective and Contagious Diseases

**Published:** 1880-10

**Authors:** W. S. Greenfield


### ART. XX. — LECTURES ON SOME RECENT INVESTIGATIONS INTO THE PATHOLOGY OF INFECTIVE AND CONTAGIOUS DISEASES.

BY W. S. GREENFIELD, M. D., LONDON, F.R.C.P.,

Professor Superintendent of the Brown Institution Assistant-Physician and Lecturer, on Pathological Anatomy to St. Thomas's Hospital.

### LECTURE I.

### PART II.

THE hypotheses as to the nature of the contagia in these two classes of disease—infective and specific—have been numerous. In the case of the infective diseases it is natural to look to the process of decomposition, and to seek either in the products or the agents of the decomposition of animal fluids or tissues the source of contagion. But, in spite of recent discoveries, opinions are divided as to whether there is any organized element having an existence independent of the putrefactive process which is the agent both of the decomposition and of the contagion.

With regard to the specific contagia, the hypotheses most commonly entertained may be classified in three main groups, and expressed in the following way :—(1) That the contagium of any specific disease consists of some protoplasmic constituent of the body possessing the power, when transferred to the body of another individual, of setting up a similar disease process ; or, (2), That an unorganized ferment produced in the disease is transferred, and sets up the vital change; or (3), That the contagium is some organism having an existence independent of the body, but capable of growing and multiplying within it, and by this growth and reproduction giving rise in some way to the phenomena of the disease. To the first and third of these hypotheses the term " germ theory" is equally applicable ; whilst to the latter alone is usually limited the term " theory of the *contagium vivum*, or *contagium animatum*."

In considering these hypotheses we must bear in mind that the contagia are not merely specific, in the sense of giving rise each to its peculiar series of changes when introduced into the body—changes which may be likened to those caused by a poison or an unorganized ferment, such as diastase or ptyaline—but that each contagium is reproduced in enormous amount in every case of infection, and that an extremely minute quantity introduced into another living body will again give rise to similar phenomena with like self-multiplication; and this cycle of change may be continued in an indefinite series under the most varying conditions. From what little we do know of the life history of contagia, especially as to their cycle of development, their reproduction and enormous self-multiplication within the body, the minute quantity needed to give rise to infection, and the absence of relation between quantity and effect, the capacity in many cases of long dormancy and resistance to some chemical and thermal influences, most pathologists have been led to reject the unorganized ferment theory as not sufficiently supported by known facts, and to accept one of the other hypotheses in preference.

To state these more fully, the first hypothesis consists in the view that a particular tissue or element of the organism having undergone some particular change or vital reaction, is capable of impressing upon another similar organism the tendency to a like vital change; in fact, that a communication of vital energy, analagous to the transmission of molecular force is the essential factor in contagion. In favor of this view is what we see in the propagation of morbid growths, or even, I might say, of normal growths in the body. For every specialised tissue element not only reproduces its like under normal conditions, but we have strong reasons to believe that the leucocytes grow into specialised elements under the influence of some of the tissues into which they enter, which thus assimilate them. In the same way morbid tissues and morbid processes repeat themselves under favoring conditions, bending the tissue elements and the nutritive processes to their own likeness, and in so doing often display a selective tendency for tissues analagous to those in which they have arisen.

The other hypothesis regards the essential contagium as consisting in some organism foreign to the body, and possessing an independent existence, which has the power of self-multiplication within the body by nutriment derived from it, and of propagating its species further by seeds or germs given off from the body. During this process of self-multiplication, changes are produced in the body, which we know as febrile and specific symptoms and lesions, which together make up the specific disease, and which upon this hypothesis may be the direct consequence of the self-multiplication of the foreign organism, or of some special fermentative change produced by it in the tissues and fluids of the body itself. This latter hypothesis is equivalent to that of the contagium vivum or contagium animatum, as defined by Dr. Burdon-Sanderson ;* and although it is not equivalent to a bacterial theory of contagion (for the organisms on which the disease depended might be of some other nature than bacteria), yet our present knowledge on the subject points towards the view that if microphytes constitute the contagia, they are probably bacterial.

I need not enter further into the discussion of this question, especially as it has been so ably dealt with by many writers. I may refer especially to the writings of Nageli, Sir Thomas Watson, Professor Lister, Dr. Maclagan, and others. But it is well known that there is at the present time a very strong tendency to the view that bacteria or some allied lower organisms are intimately related to contagion, either as the actual contagium, or as carriers of it, or as an essential condition of its potency. The

* Dr. Sanderson has thus expressed this view; "According to the view which these words (contagium vium) are understood to express, the morbific material by which a contagious disease is communicated from a diseased to a healthy person consists of minute organisms called 'disease germs.' In order that any particle may be rightly termed a disease germ, two things must be proved concerning it—namely, first, that it is a living organism; secondly, that if it finds its way into the body of a healthy human being, or of an animal, it will produce the disease of which it is the germ." (Proc. Roy. Soc., Nov. 22d, 1877, No. 184, p. 122). In quoting this, I do not wish to express agreement in so strict a limitation of the words "disease germs."

precise relation of bacteria to contagion has long been a favorite subject of speculation, and every new fact ascertained with regard to their development seems to be hailed or rejected by some persons, according as it appears to favor or oppose some theory upon this subject. At present, therefore, I shall content myself with pointing out that in what we now know of the life history of certain bacterial forms there is some presumptive evidence in favor of their being the actual contagia in certain diseases. For in the first place, of all known organisms, bacteria most closely conform in their life conditions to what we know of specific contagia, as to nutrition, rapidity of multiplication, powers of latency, and retention of vitality in the form of spores. Indeed, recent discoveries as to the life conditions and cyclic changes of bacilli show a most striking analogy with certain contagia. And, secondly, in certain diseases, notably splenic fever and contagious pneumo-enteritis of swine, it may be regarded as conclusively proved that the contagium is a bacillus, and we have thus some positive evidence in favor of the hypothesis.

Turning now to the subject of bacteria, we find that, important as are the discoveries which have been made, we must be content with a knowledge as to their mutual relations, life history, and variations under cultivation, which is, perhaps, more confusing and difficult than the subject of contagion itself. In the present transitional state of our knowledge we must anticipate conflicting views, and it need hardly surprise us that diametrically opposite opinions are entertained by those whose special study and acquaintance with the subject lend weight to their authority. Let me mention one or two of the more important of these disputed points. Are there a multitude of different bacterial forms, each separate from the other in its origin, its action, and its form; or, rather, its life history? Or are all the variations in form and size, in development, and, what is more important, in reaction, merely phases in the development of one protean form? Does every change in condition of life, every fermentative process, lead to a modification of this original form, and the acquisition of special powers of reaction, which are capable of transmission by heredity, so long as favoring conditions exist, but which die away when those conditions cease, to be called again into existence and potency when like conditions recur. In favor of some such view I may quote two distinguished authorities. Nageli, one of the earliest workers in this field, has said that he has never seen reason to believe in the existence of more than two kinds of bacteria. Professor Ray Lankester holds, I believe, the view that only bacilli are separate from common bacteria, the latter being mutable, with varying conditions. As a contrast to this I may mention that Koch* believes that all bacteria (pathological at any rate) are bacilli, holding firmly, however, that there are a large number of absolutely distinct pathological bacteria, not only distinguishable by their size and form, but by their special physiological and pathological reactions. Professor Cossar Ewart has done good work in showing how closely the several morphological varieties or classes may approach one another in certain stages of their existence.

I might quote many distinguished names in support of one or other of these more or less modified views, but time forbids. I believe that for practical purposes it is well that we should hold provisionally, as a probable and workable hypothesis, that there are many distinct kinds of bacteria, that they are probably distinguishable either by their physical characters or their vital changes, or by some power which they possess. And we may leave the solution of the metaphysical question, whether they originated from some common stock, and have merely acquired certain potencies and forms, until we know more of their actual relation to the processes in which they are concerned.

Seeing, then, how important is a knowledge of the various forms and phases of bacteria, in order to a comprehension of their relation to putrefactive and contagious processes, I trust I may be excused if I venture to give a brief and elementary account of some of the more important of the common forms, which may be of use to those who have not specially studied the subject; and in doing so I shall refer almost exclusively to such facts as have some probable bearing on the study of disease.

---

* Klebs Archiv. f. Path.

Disregarding for the moment the nature, habit, size, and so on, of bacteria, we may roughly divide them, following Cohn, into spheroidal bacteria, typified by micrococcus; rod-shaped bacteria, of which the type is bacterium termo; long-rod bacteria or bacilli; and spiral or twisted bacteria, of which the type is spirillum. We now know that in the course of development of bacteria and bacilli, short and long rod forms, and also more or less spherical forms, are produced, so that mere form in one stage does not serve as a ground of distinction; but we have as yet reason to doubt whether some bacteria ever pass beyond the spherical stage, whilst others do not appear to develop into elongated rods; others, again, habitually assume rod or spiral forms.

It would occupy far too much time to enter with any detail into the various researches into the mode of development of bacteria, or even to mention the numerous workers in this field. The observations of Ehrenberg, Nageli* Cohn,† Pasteur, Klebs,‡ Billroth,|| Dallinger,§ Huxley, Sanderson, Lister, Ray Lankester,¶ Koch,** Klein,†† Cossar Ewart,‡‡ Lewis,|||| and many others, are familiar to all who have made a study of the subject. As a very valuable elementary help in the consideration of many difficult points, I may mention the papers by Dr. Cossar Ewart, the accuracy of whose descriptions I can for the most part confirm from personal observation.

---

* Nägeli, Die Niederen Pilze.

† Cohn, various papers in Beiträge zur Biologie d. Pflanzen, Band i., Heft 2, p. 127, and Heft 3, p. 141; Band ii., p. 249.

‡ Klebs, various papers in Achiv. f. Pathologie.

|| Billroth, Coccobacteria Septica.

§ Dallinger, various papers in Phil. Trans., Journal of Royal Microscopical Society, etc.

¶ Ray Lankester, various papers in Proc. Royal Soc., Quarterly Journal of Microscopical Science, and elsewhere.

** Koch, Beit zur Biol. d. Pflanzen, Band ii., p. 277, and Band ii., p. 399.

†† Klein, various papers in Quart. Jour. Micr. Science, Proc. Roy. Soc., and Seventh Annual Report of Med. Officer of Loc. Gov. Board.

‡‡ Cossar Ewart, Microsc. Jour., vol. xviii., and Proc. Roy. Soc., No. 188, 1878.

|||| Lewis, Dr. Timothy, Microscopic Organisms found in the Blood in Relation to Disease.

In the following brief sketch I shall limit myself to the more essential facts with regard to the commoner orders—Micrococcus, bacterium, and bacillus—and chiefly state those points which are matters of personal observation.

GENERAL CHARACTERS OF BACTERIA.

*Structure.*—Bacteria have been defined by Cohn as cells free from chlorophyll, of spherical, oblong or cylindrical, sometimes twisted or curved form, which increase by transverse division exclusively, and grow either isolated or in cell families. These cells consist of protoplasmic, usually colorless, cell contents, strongly refractile, in which usually shining, oil-like nucleoli or nuclei are imbedded. The protoplasm is flexible or contractile; hence their spontaneous bending and stretching when the membrane is not too rigid. The protoplasm having a different refractive index from that of water and most fluids in which they grow, their production in abundance gives rise to a milky appearance. This protoplasm is surrounded by a cell membrane, shown both by its resistance to reagents and by actual observation; this is composed of cellulose or some analogous substance. Cohn believes that the membrane is soluble, and can form the intercellular substance.

The division of bacteria, the characteristic mode of reproduction, is accomplished by longitudinal stretching to a length nearly double the normal; then a transverse constriction appears at the centre, and sooner or later the two members separate, and this process continues indefinitely. Longitudinal division is, according to Cohn, extremely rare in free bacteria. In some forms of bacteria the individual members remain attached, then form long chains, especially under certain conditions; but when free, and in most forms, they rapidly separate, only two, three, or four elements remaining attached. In most cases, when the bacteria elongate to form long threads, the division may be invisible, or seen only with difficulty.

Under certain conditions, sperical and rod bacteria, instead of elongating and dividing, and forming free single or double elements, become agglomerated into a mass consisting of a large number of individuals united by a sort of intercellular substance, which form rounded, or flattened, or irregular masses in the nutrient fluid. In these masses the bacteria continue to multiply, the individual elements being closely massed together at first, but by increase of the intercellular substance they may become more widely separated. These zoogl∙a masses are liable to absorb coloring matter, or to be stained by iron, or by iron and sulphur, if contained in the fluid. The multiplication in zoogl∙a having continued to the formation of an immense number, these may become broken up by solution of the intercullular substance, and the elements become free in the fluid. According to Cohn, this zoogl∙a formation is never observed either in filamentous or twisted bacteria (bacillus or spirillum) though they may accumulate in masses.

When bacteria are growing in any fluid they are apt to accumulate more or less on the the top, and form a layer on the surface, called by Pasteur mycoderma, this being distinguished, according to Cohn, from zoogl∙a by being composed merely of individual elements without intercellular substance.

The formation of spores of all forms of bacteria is as yet undecided. Cohn now believes that only bacilli form spores. But in zoogl∙a masses one constantly finds ovoid or rounded bodies, apparently derived from bacteria, and often, too, one sees bacteria containing nuclei resembling the spores of bacilli.

Most bacteria have a motile and non-motile condition, and these alternate. Some bacteria—e.g., bact. termo—are usually motile, others motionless. In a certain number the movement has been shown to depend on cilia, in others evidence of their presence is as yet wanting.

Lastly, bacteria require the presence of oxygen for their growth and development, but are not absolutely destroyed by its absence.

Such, in brief, are some of the more important facts with regard to the nature of bacteria.

The *classification* of *schizomycetes* or *schizophytes* proposed by

Cohn in 1872,* to which I have already referred, may serve as a convenient basis of description, although many distinctions which formed part of the groundwork of his classification, cannot now be maintained. It is as follows:

Tribe I. Sphæro-bacteria (Kugel-bacterien).
Group 1. Micrococcus (char. emend).
Tribe II. Micro-bacteria (Stäbchen-bacterien).
Group 2. Bacterium (char. emend).
Tribe III. Desmo-bacteria (Faden-bacterien).
Group 3. Bacillus.
" 4. Vibrio (char. emend).
Tribe IV. Spiro-bacteria (Schrauben-bacterien).
Group 5. Spirillum (Ehrenberg).
" 6. Spirochæte (Ehrenberg).

